# Differentiation of peripheral sensory neurons from iPSCs derived from stem cells from human exfoliated deciduous teeth (SHED)

**DOI:** 10.3389/fcell.2023.1203503

**Published:** 2023-07-13

**Authors:** Nathalia C. Oliveira, Fabiele B. Russo, Patricia C. B. Beltrão-Braga

**Affiliations:** ^1^ Disease Modeling Laboratory, Department of Microbiology, Institute of Biomedical Sciences, University of São Paulo, São Paulo, Brazil; ^2^ Neurobiology Laboratory, Scientific Platform Pasteur-USP, São Paulo, Brazil

**Keywords:** iPSC differentiation, peripheral nervous system, peripheral sensory neuron, neural crest cell, SHED

## Abstract

Peripheral nervous system (PNS) sensory alterations are present in several pathologies and syndromes. The use of induced pluripotent stem cell (iPSC) technology is an important strategy to produce sensory neurons in patients who are accomplished in terms of sensory symptoms. The iPSC technology relies on manipulating signaling pathways to resemble what occurs *in vivo*, and the iPSCs are known to carry a transcriptional memory after reprogramming, which can affect the produced cell. To this date, protocols described for sensory neuron production start using iPSCs derived from skin fibroblasts, which have the same ontogenetic origin as the central nervous system (CNS). Since it is already known that the cells somehow resemble their origin even after cell reprogramming, PNS cells should be produced from cells derived from the neural crest. This work aimed to establish a protocol to differentiate sensory neurons derived from stem cells from human exfoliated deciduous teeth (SHED) with the same embryonic origin as the PNS. SHED-derived iPSCs were produced and submitted to peripheral sensory neuron (PSN) differentiation. Our protocol used the dual-SMAD inhibition method, followed by neuronal differentiation, using artificial neurotrophic factors and molecules produced by human keratinocytes. We successfully established the first protocol for differentiating neural crest and PNS cells from SHED-derived iPSCs, enabling future studies of PNS pathologies.

## 1 Introduction

The use of induced pluripotent stem cells (iPSCs) in disease modeling and drug screening has been widely spread in biomedicine (Omole & Fakoya, 2018) but mainly as a tool for the study of neurodegenerative and neurodevelopmental disorders probably because it somehow allows access to the central nervous system (CNS), permitting the exploration of molecular pathways of certain subtypes of active human neurons and other neural cells (Marchetto et al., 2011; Russo et al., 2018). Additionally, iPSCs keep the genetic background of the donor, which is especially important to study polygenic and multifactorial disorders like autism spectrum disorder (ASD) (Marchetto et al., 2009). Human iPSCs have been used to generate several neuronal and glial subtypes (Tcw et al., 2017; Bianchi et al., 2018; Jerber et al., 2021; Johns & Maragakis, 2022; Xu et al., 2022), as well as whole-brain organoids (Lancaster et al., 2013) or organoids from specific brain regions (Qian et al., 2018; Jacob et al., 2020), to develop a basis for understanding neurological diseases.

Despite the numerous advances in iPSC-derived nerve cells, there are few protocols to produce sensory neurons, which are the cells that capture environmental or visceral stimuli and transmit them to the CNS (Chambers et al., 2012; Eberhardt et al., 2015; Bear et al., 2016; Guimarães et al., 2018; Schwartzentruber et al., 2018; Mis et al., 2019; Umehara et al., 2020). In addition, sensory neurons have recently been described as very much affected during viral infections, like in the coronavirus disease of 2019 (COVID-19) pandemic, in which 80% of infected people manifested alterations in senses related to the peripheral nervous system (PNS), like ageusia (loss of sense of taste) and anosmia (loss of sense of smell) (Cooper et al., 2020; Patel et al., 2020; Cunhados et al., 2021). Once again, sensorial stimuli seem to be affected in neurodevelopmental disorders, such as ASD and attention-deficit hyperactivity disorder (ADHD), in neuropathic pain, and specific sensory disorders, like sensory processing disorder (SPD) (Koziol et al., 2011; Weiland et al., 2011; Hazen et al., 2014; Labau et al., 2022).

Neurons from the sensory system still face difficulties in differentiation protocols, which impact their use for *in vitro* modeling (Labau et al., 2022). Sensory neuron differentiation protocols (Chambers et al., 2012; Eberhardt et al., 2015; Guimarães et al., 2018; Schwartzentruber et al., 2018; Mis et al., 2019; Nickolls et al., 2020; Umehara et al., 2020) have been tested on more than 100 different human iPSC (hiPSC) strains, all derived from pulmonary, dermal fibroblasts, or blood, which are cells of mesenchymal origin (LeBleu & Neilson, 2020). It is important to consider that sensory neurons belonging to the PNS originate in the neural crest, derived from ectoderm differentiation during the neurulation process (Huang & Saint- Jeannet, 2004). The neural crest cells act in the formation of several parts of the vertebrate body: in the formation of pigmented cells, originating the ganglia of the peripheral nervous system and composing part of the cartilages and bones of the face; in the cardiac formation (cells that form septa and valves, and muscle cells); and in tooth structure, originating cells forming dentin and, in mammals, pulp, cementum, periodontal ligament, and alveolar bone (Hall, 2009; Szobó and Mayor, 2018). In the pulp of human exfoliated deciduous teeth, there is a population of postnatal multipotent stem cells called stem cells from human exfoliated deciduous teeth (SHED), which have a high proliferation rate and the ability to form clusters, and are osteoinductive *in vivo* (Miura et al., 2003). Because they share the same embryonic origin as some neural cells, SHED express neuronal and glial markers (Miura et al., 2003), and the dental pulp cells produce neurotrophic factors and interact with peripheral nervous system neurons *in vitro* (Nosrat et al., 2001). Considering that even after reprogramming hiPSCs maintain a particular transcriptional signature from the donor cell (Marchetto et al., 2009), it is essential to choose for reprograming the cell that is derived from the same germ layer as the final differentiated cell, which is the target of the study. Here, we have established a protocol to obtain functional peripheral sensory neurons (fPSNs) derived from SHED to use as a platform for studying sensory alterations or diseases that affect this system.

## 2 Materials and equipment

**Table:** 

Reagent	Source	Identifier
**SHED isolation and culture**		
Phosphate-buffered saline (PBS) 0.01 M, pH 7.4	Sigma	*Cat #P4417-100TB*
Collagenase enzyme type 1	Gibco	*Cat #17100–017*
Penicillin–streptomycin (10,000 U/mL)	Gibco	*Cat #15140122*
Dulbecco’s modified Eagle’s medium (DMEM)/Ham’s F12 (1:1)	Thermo Fisher Scientific	*Cat #*11330057
Fetal bovine serum (FBS)	LGC	*Cat #10-bio500*
L-glutamine	Invitrogen	*Cat #25030081*
Non-essential amino acids	Gibco	*Cat #11140050*
TrypLE™ select enzyme	Gibco	*Cat #12563011*
**iPSC reprogramming**		
Sendai virus (CytoTune^®^)	Thermo Fisher Scientific	*Cat #*A16517
Dulbecco’s modified Eagle’s medium (DMEM)/Ham’s F12 (1:1)	Invitrogen	*Cat #*11330057
Non-essential amino acids	Gibco	*Cat #11140050*
Knockout serum replacement (KOSR)	Gibco	*Cat #*10828–028
Murine embryonic fibroblasts (MEFs)	Millipore	Cat#PMEF-CF-C
FGF-2	Thermo Fisher Scientific	Cat# PHG0261
**iPSC culture feeder-free**		
mTeSR	Stem Cell Technologies	*Cat #*05850
Matrigel	BD. Biosciences	*Cat #356230*
**fPSN differentiation**		
Dulbecco’s modified Eagle’s medium (DMEM)/Ham’s F12 (1:1)	Gibco	*Cat #11330057*
Neurobasal medium	Gibco	*Cat #21103049*
N2 Supplement 100X	Gibco	*Cat #17502048*
B27 Supplement 50X	Gibco	*Cat #17504044*
GlutaMAX 100X	Gibco	Cat #*35050061*
MEM-NEAA 100X	Gibco	*Cat #11140050*
Beta-mercaptoethanol 1000X–55µM	Gibco	*Cat #21985023*
Penicillin–streptomycin (10,000 U/mL)	Gibco	*Cat #15140122*
LDN-193189	Sigma-Aldrich	*Cat# SML0559*
SB431542	Tocris	*Cat# 1614*
CHIR-99021	Tocris	*Cat# 4423*
FGF-2	Thermo	*Cat# PHG0263*
EGF	Thermo	*Cat# PH0313*
Poly-L-ornithine	Sigma	*Cat# P3655-100MG*
Laminin	Gibco	*Cat#23017015*
Accutase	Gibco	*Cat#A1110501*
BDNF	R&D	*Cat#248-BDB-010*
Ascorbic acid (AA)	Sigma	*Cat#A8960*
GDNF	R&D	*Cat#212-GD-010*
NGF	R&D	*Cat#256-GF-100*
NT-3	R&D	*Cat#267-N3-005*
cAMP	Sigma	*Cat#D0627*
Fetal bovine serum (FBS)	LGC	*Cat #10-bio500*
Dimethylsulfoxide (DMSO)	LGC	*Cat# BR2650-01*
**HEK culture**		
KGM medium	Lonza	*Cat #11635420*
**Immunocytochemistry materials**		
Paraformaldehyde (PFA)	Labsynth	*Cat#01P1005.01.AH*
Triton X-100	Sigma-Aldrich	*Cat#X100*
Bovine serum albumin (BSA)	GeminiBio	*Cat#700–110*
DAPI	Invitrogen	*Cat#D1306*
ProLong Gold Antifade Reagent	Thermo Fisher Scientific	*Cat#P10144*
Anti-OCT4 antibody	Abcam	*Cat#ab18976*
Anti-NANOG antibody	Abcam	*Cat#Ab77095*
Anti-SOX2 antibody	Cell Signaling Technology	*Cat# 2748*
Anti-nestin antibody, mouse	Abcam	*Cat#ab22035*
Anti-peripherin antibody, rabbit	Abcam	*Cat#ab246502*
Anti-synapsin I antibody, rabbit	Abcam	*Cat#ab1543*
Anti-MAP2 antibody, chicken	Abcam	*Cat#ab92434*
Anti-islet1 antibody, mouse	Abcam	*Cat#ab86501*
Goat anti-mouse, Alexa Fluor 488	Thermo Fisher Scientific	*Cat#A28175*
Goat anti-rabbit, Alexa Fluor 555	Abcam	*Cat#ab150078*
Goat anti-chicken, Alexa Fluor 488	Abcam	*Cat#ab150169*
Goat anti-mouse, Alexa Fluor 555	Thermo Fisher Scientific	*Cat#ab150114*
**Equipment and software**		
CO_2_ incubator	Thermo Fisher Scientific	*Cat#4120*
Biological safety cabinet	Thermo Fisher Scientific	*Cat#1323TS*
60-mm tissue culture dish	Jet Bio-Filtration Co.	*Cat#TCD010060*
100-mm tissue culture dish	Jet Bio-Filtration Co.	*Cat#TCD010100*
24-well tissue culture dishes	Jet Bio-Filtration Co.	*Cat#TCP011024*
Micropipette monochannel (1000 µL)	FirstLab	*Cat#K1-1000B*
Micropipette monochannel (200 µL)	FirstLab	*Cat#K1-200B*
Micropipette monochannel (20 µL)	FirstLab	*Cat#K1-20B*
Micropipette monochannel (2 µL)	FirstLab	*Cat#K1-2B*
Filter pipette tips (100–1000 µL)	Jet Bio-Filtration Co.	*Cat#PPT101000*
Filter pipette tips (10–200 µL)	Jet Bio-Filtration Co.	*Cat#PPT151200*
Filter pipette tips (2–20 µL)	Jet Bio-Filtration Co.	*Cat#PPT101020*
Filter pipette tips (0.1–10 µL)	Jet Bio-Filtration Co.	*Cat#PPT101010*
Sterile glass bottle (250 mL)	Laborglas LTDA	*Cat#91801365*
Sterile tubes (15 mL)	KASVI	*Cat#K19-0015*
Automatic pipette	KASVI	*Cat#K1-AID-B*
25-mL serological pipettes	Jet Bio-Filtration Co.	*Cat#CSP010025*
Cryogenic vials (2 mL)	Corning	*Cat#430659*
Benchtop centrifuge	Centrilab	*Cat#80-2B*
37°C water bath	Thermo Fisher Scientific	*Cat#TSGP10*
4°C refrigerator	Thermo Fisher Scientific	*Cat#TSG505GA*
Glass cover slip	Olen	*Cat#K5-0013*
Glass slide	FirstLab	*Cat#FL6-7105-1*
Microcentrifuge tubes (2 mL)	Jet Bio-Filtration Co.	*Cat#CFT-000–020*
Sterile clamp	ABC Instrumentais	*Cat#0554*
EVOS XL core imaging system	Thermo Fisher Scientific	*Cat#AMEX1000*
AMG EVOS FL digital inverted microscope	Thermo Fisher Scientific	*Cat#AMF-4301*
LEICA DMi8 wide-Field microscope	Leica Microsystems	
LAS X software	Leica Microsystems	
ImageJ software	National Institutes of Health	

## 3 Methods

### 3.1 Ethics statement, human samples, and primary cell culture

This project was approved by the ethics committee (CEP 4.144.470). Subjects were recruited through The Tooth Fairy Project initiative (University of São Paulo—USP), with approval by the Ethics Committee of the Institute of Biosciences CEP- ICB/USP (Protocol CEP/ICB-USP 1001, Biorepository: CAAE 58219416.0.0000.5467). After a complete description of this research, parents were provided with informed consent for their participation in donating the deciduous tooth after the exfoliation. Dental pulp was extracted from the tooth, and the tissue was washed twice using sterile phosphate-buffered saline (PBS; 0.01 M, pH = 7.4) supplemented with 5% antibiotics (500 U/mL penicillin and 500 μg/mL streptomycin, Sigma). After washing, collagenase enzyme type 1 (Gibco, Life Technologies) was used for tissue matrix digestion isolating cells. SHED were cultivated with Dulbecco’s modified Eagle’s medium (DMEM)/Ham’s F12 (1:1, Invitrogen) supplemented with 15% fetal bovine serum (FBS), 1% penicillin–streptomycin, 1% glutamine, and 1% non-essential amino acids. Cells were passed every 4–5 days with the medium being refreshed daily. All cultures were incubated at 37°C with 5% CO_2_ in a high-humidity environment.

### 3.2 iPSC generation and maintenance

Cellular reprogramming experiments (Beltrão-Braga et al., 2011; Russo et al., 2018) were conducted using the Sendai virus (CytoTune^®^ - Gibco, Life Technologies) carrying genetic material from Yamanaka’s factors (Oct4, Sox2, Kfl4, and c-Myc) (Takahashi et al., 2007). SHED were transduced, and after 2 days, the cells were transferred to a feeder-layer condition consisting of murine embryonic fibroblasts (MEFs, Millipore) and maintained with DMEM/F12 (Gibco, Life Technologies), 20% KnockOut Serum Replacement (Invitrogen), 1% non-essential amino acids, 100 μM beta-mercaptoethanol, and fibroblast growth factor 2 (FGF2) (30 ηg/mL). The iPSC colonies, identified after approximately 3 weeks, were transferred to Matrigel (BD Biosciences)-coated plates. Once iPSCs were on feeder-free plates, they were maintained in mTeSR media (Stem Cell Technologies) and changed daily. These cells underwent G-banding karyotype performed at Children’s Hospital of Los Angeles, CA, to confirm if all isolated clones maintained the normal karyotype after reprogramming, and all abnormal karyotype cell lines were discarded (Russo et al., 2018).

### 3.3 Conditioned medium for human epidermal keratinocyte culture

The human epidermal keratinocytes (HEKs), foreskin-originated, were cultured in KGM medium (Lonza), and when the plate reached 70% confluence, the keratinocyte medium was changed to 3 N media, which was described in the following section, and maintained for 48 h for conditioning. The conditioned medium was centrifuged before use to remove debris, and it was used in the final stage of the protocol for the generation of fPSN.

### 3.4 Functional peripheral sensory neuron differentiation

The differentiation protocol to produce fPSNs was based on Guimarães et al. (2018). The medium used for fPSN differentiation was 3 N medium: to prepare 100 mL of the medium, combine 48.5 mL of DMEM-F12 (Gibco), 48.5 mL of the Neurobasal medium (Gibco), 1 mL of Glutamax (100x; Gibco), 1 mL of non-essential amino acids (NEAAs) (100x; Gibco), 1 mL of N2 supplement (100x; Gibco), 2 mL of B27 supplement (50x; Gibco), 90 µL of beta-mercaptoethanol (1000x; Gibco), and 1 mL of penicillin–streptomycin (10,000 U/mL; Gibco).

When the iPSC reached approximately 60% confluence with small colonies (∼500µm in diameter), the mTeSR medium was changed to a 3 N medium supplemented with 500 nM of LDN-193189 and 10 µM of SB431541 for 24 h. After this period, 3 µM of CHIR-99021 and the other supplements were added. The next day, LDN-193189 was removed, and after another 24 h, SB431541 was also removed, leaving only CHIR-99021 for another 6 days, with media being changed every 2 days. After this period, the cells were transferred using Accutase diluted in 1 × PBS (1:1) to 6-cm plates treated with poly-L-ornithine (10 μg/mL) and laminin (2.5 μg/mL) (PO-lam). From this stage, they were named neural crest progenitor cells (NCPCs) and were cultured with a 3 N medium supplemented with 10 ng/mL of FGF2 and 10 ng/mL of epidermal growth factor (EGF) for expansion and stock. NCPC expansion was performed by adding Accutase to the plate and leaving it for 5 min in an incubator at 37°C to detach. Twice as much medium was added to the Accutase–cell mixture to stop the reaction, and the cells were centrifuged for 5 min at 1000 rpm. Cells were re-suspended in a 3 N medium supplemented with FGF and EGF, and plated on a PO-lam-treated plate. When the NCPC reached approximately 80% of confluence, cells were seeded in a 24-well plate treated with PO-lam using the same medium and supplements described previously. The differentiation of peripheral sensory neurons (PSNs) started 24 h after seeding. For this procedure, the FGF and EGF were removed, and other factors such as 10 ng/mL brain-derived neurotrophic factor (BDNF), 200 µM ascorbic acid, 10 ng/mL glial cell line-derived neurotrophic factor (GDNF), 10 ng/mL nerve growth factor (NGF), 10 ng/mL neurotrophin-3 (NT-3), and 0.5 mM cyclic adenosine monophosphate (cAMP) were added. This condition was maintained for 20 days with a media change every 5 days, and, in the end, the PSNs were obtained.

After this period, the PSN cultures were maintained with 75% of the conditioned medium on HEK and 25% of the new 3 N medium for another 10 days to mature and obtain fPSNs. All six supplements were also added in this stage.

### 3.5 Cellular characterization by immunofluorescence assay

Cells were fixed in 4% paraformaldehyde (PFA) for 15 min at room temperature (RT) and permeabilized with 0.1% Triton X-100 for 15 min at RT. Fixed cells were blocked with 2% bovine serum albumin (BSA) (Sigma-Aldrich) for 4 h at RT and then incubated overnight with the primary antibodies (Table 1) at 4°C. The following day, cells were washed thrice with dPBS and blocked again for 1 hour with 2% BSA at RT. Secondary antibodies, such as goat anti-mouse Alexa Fluor 488 (Thermo Fisher Scientific), goat anti-rabbit Alexa Fluor 555 (Abcam), mouse anti-goat Alexa Fluor 555 (Abcam), goat anti-chicken Alexa Fluor 488 (Abcam), and goat anti-mouse Alexa Fluor 555 (Thermo Fisher Scientific), at 1:500 were added for 1 hour at RT. Cells were triple-washed with dPBS, and nuclei were stained using DAPI (Invitrogen) at 1:10,000 diluted in a 1x dPBS solution for 5 min. Cells were washed once after the DAPI addition with dPBS and mounted using the ProLong Gold Antifade Reagent (Invitrogen). Images were acquired using the AMG EVOS FL digital inverted microscope (Thermo Fisher Scientific.) and the LEICA DMi8 wide-field microscope (Leica Microsystems).

**TABLE 1:** Antibodies used in this study related to experimental procedures.

Antibody	Manufacturer	Catalog#	Dilution
Sox2	Cell Signaling Technology	2748	1:500
Oct4	Abcam	ab18976	1:100
Nanog	Abcam	ab77095	1:100
Nestin	Abcam	ab22035	1:250
MAP2	Abcam	ab92434	1:1000
Synapsin 1	Abcam	ab1543	1:200
Islet1	Abcam	ab86501	1:250
Peripherin	Abcam	ab246502	1:250

### 3.6 Immunofluorescence quantification and statistics

Image processing was performed using ImageJ and LASX software applications (Leica Microsystems). The quantification of synapsin and peripherin was obtained by LASX software, and the calculation of the relative fluorescence intensity (RFI) was performed by dividing the fluorescence intensity (F(t)) of each image by the fluorescence of the nuclear marker (F(0)), subtracting their respective background fluorescence (Fb). (RFI = [F(t)-Fb(t)]/[F(0)-Fb(0)]). Graphical and statistical analyses were based on the RFI of 20 images and were performed using Prism software (GraphPad), applying an unpaired *t*-test with Welch’s correction.

## 4 Results

**FIGURE 1:**
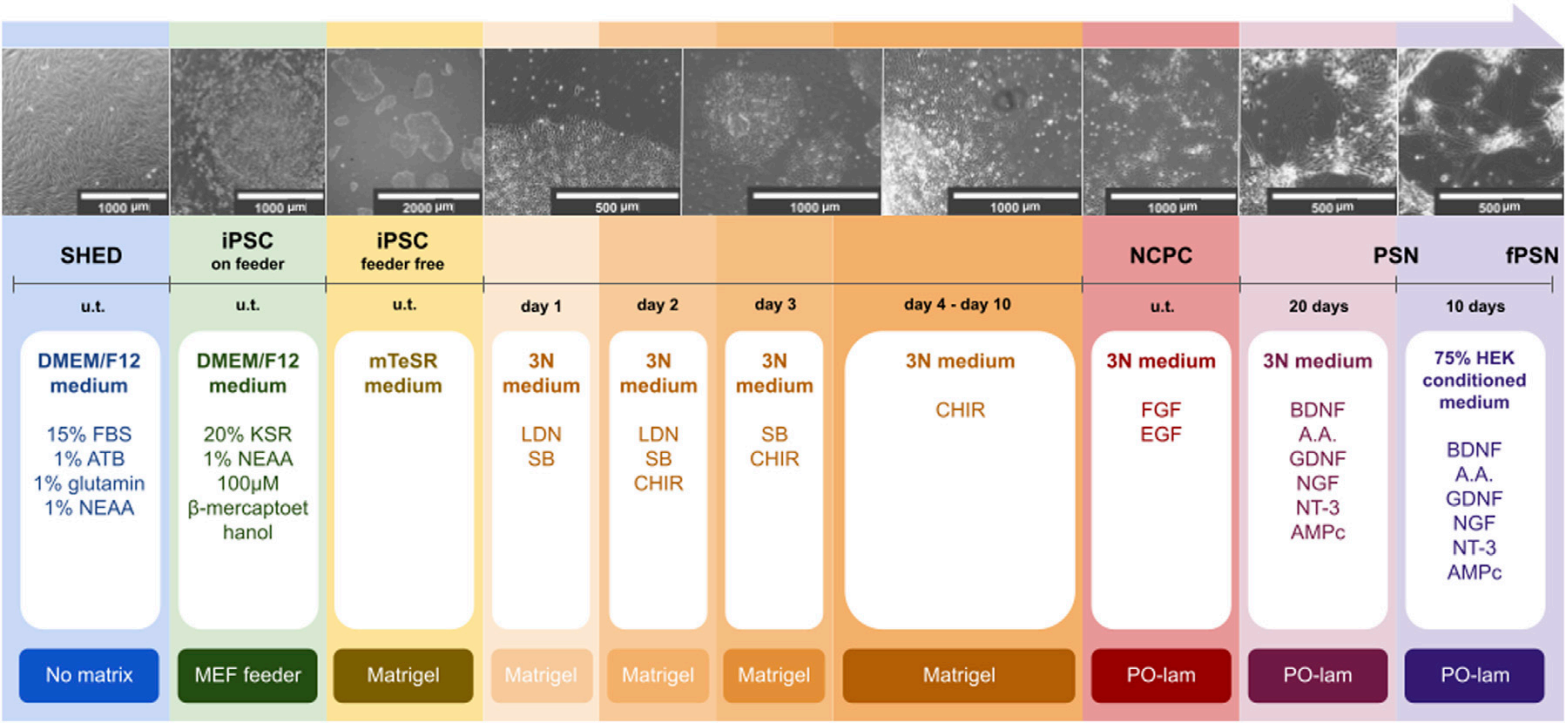
Schematic representation of the sensory neuron differentiation protocol. SHED were isolated from neurotypical individuals. Cells were reprogrammed into iPSCs using Sendai virus (u.t., undetermined time). The production of fPSNs was based on a previously described protocol (Guimarães et al., 2018). Scale bars: 2000, 1000, and 500 µm.

Our group was the first to establish the protocol to produce fPSNs derived from SHED-iPSC. fPSNs were produced based on a protocol previously established for iPSCs derived from fibroblasts (Guimarães et al., 2018). The protocol is represented in Figure 1.

**FIGURE 2:**
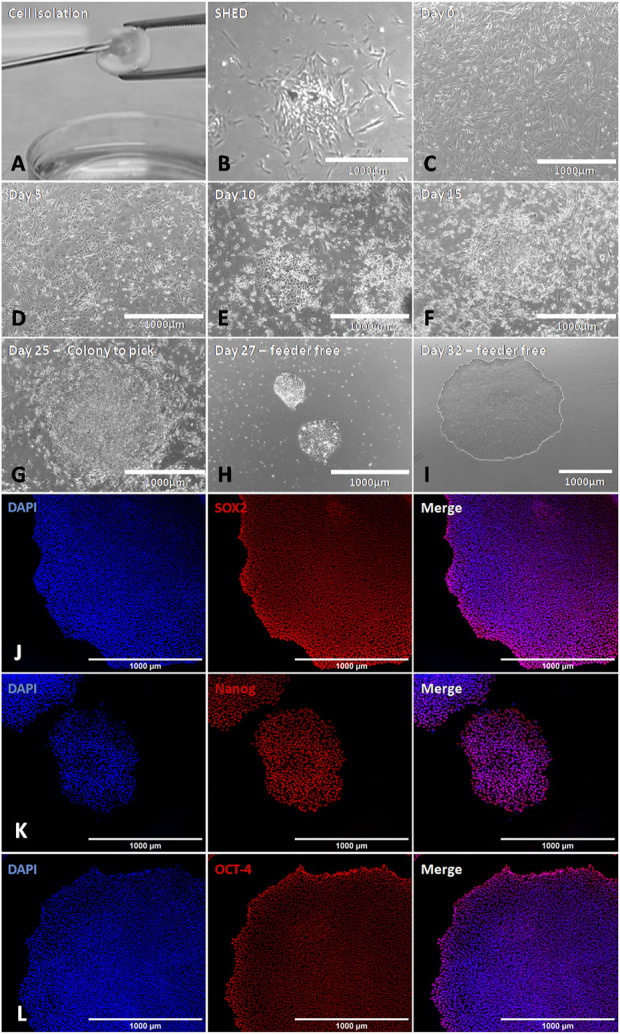
Generation of iPSCs derived from SHED using Sendai virus. **(A,B)** Representative images of SHED isolation and primary culture. **(C)** SHED before the virus infection with 1 × 10^5^ cells (day 0). **(D–F)** SHED-iPSCs cultivated on MEF (days 5, 10, and 15 after transduction). We can observe the aspect of iPSC colonies growing on the feeder. **(G)** SHED-iPSCs on MEF 25 days after transduction. At this moment, the colony is ready to pick. **(H,I)** iPSC colonies on the feeder-free condition. **(J–L)** iPSCs showed expression for Sox2, NANOG, and Oct4. Scale bar: 1000 µm.

SHED were obtained from neurotypical individuals. Cellular reprogramming was highly effective, with small iPSC colonies being observed 25 days after transduction (Figures 2A–G), which were collected and established in feeder-free culture conditions (without a supporting cell layer) (Figure 2H). The iPSC colonies showed a delimited border with a phase-bright center, and the cells had a prominent nucleus and less cytoplasm (Figure 2I). The expression of pluripotent cell markers (Sox2, NANOG, and Oct4) was analyzed by immunofluorescence assays, confirming the success of cell reprogramming (Figures 2J–L).

**FIGURE 3:**
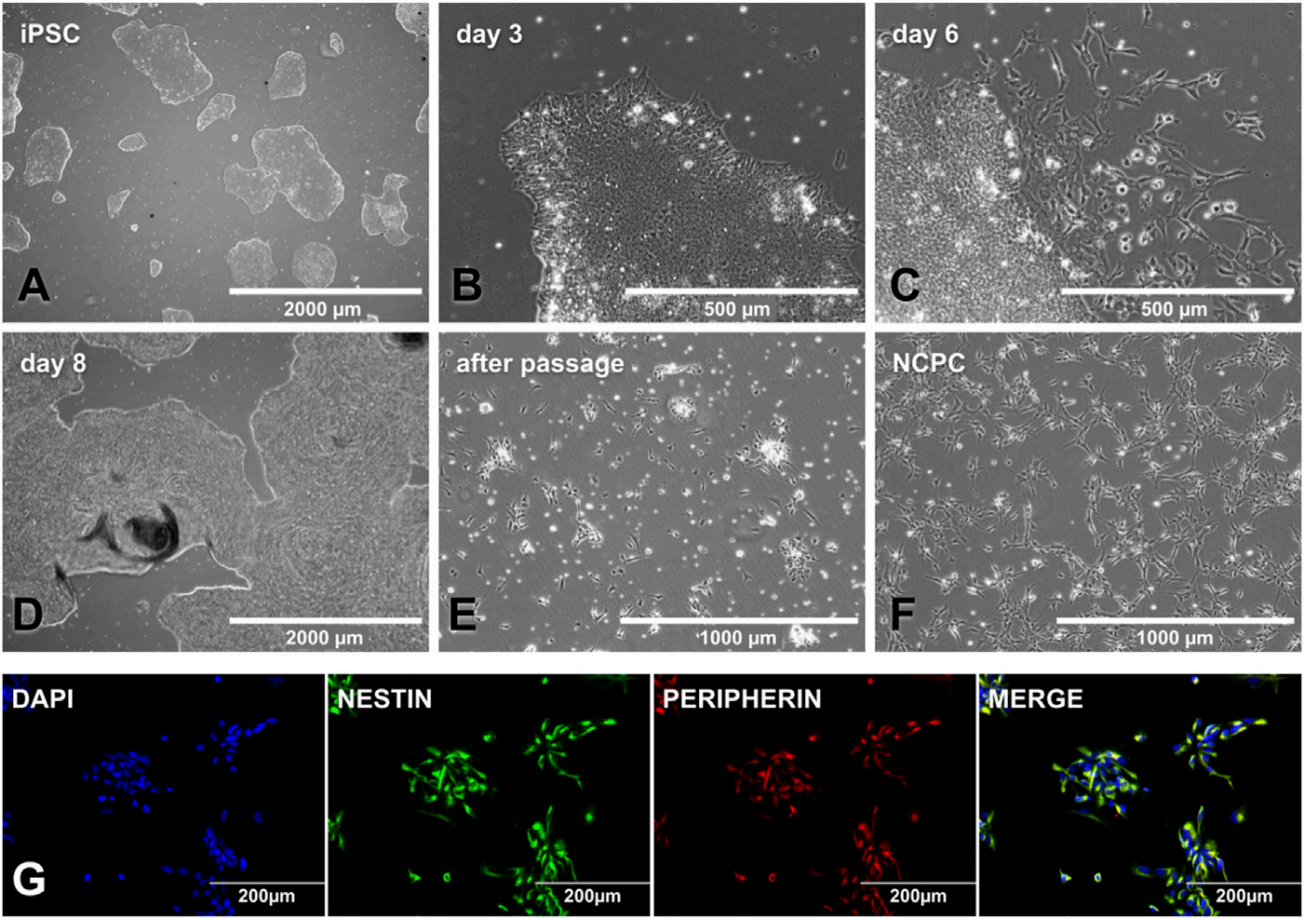
NCPC differentiation and characterization. **(A)** SHED-iPSC colonies ready to start differentiation, with a colony size of approximately 500 μm. **(B)** Day 3 NCPC differentiation, with the stretched cells in the colony edge. **(C)** Day 6 NCPC differentiation, when the cells start migrating from the colony. **(D)** Day 8 NCPC differentiation, where colonies may have overgrowth. **(E)** Cells as soon as they are transferred to the PO-lam plate, with some cellular clusters. **(F)**. NCPC-established culture. **(G)**. NCPC-expressed nestin (the neuroprogenitor marker) and peripherin (peripheral nervous system cell intermediate filament protein). Scale bar: 2000 µm **(A and D)**, 1000 µm **(E, F)**, 500 µm **(B, C)**, and 200 µm **(G)**.

The iPSC colonies must be small (∼500µm) to start the NCPC differentiation (Figure 3A) since this makes the factors reach the colony’s interior and prevents them from growing uncontrollably until day 11. Around day 3, the colony’s border is no longer delimited, and it becomes possible to observe cytoplasmic expansions on it (Figure 3B). When the culture is only supplemented with CHIR-99021, on day 6, some cells begin to migrate out of the colonies (Figure 3C), and on day 8, colonies may show overgrowth and an increased risk of detachment (Figure 3D). After transferring the cells to PO-lam-treated plates, it may take a few days to become established, and cells can form cell aggregates at this stage (Figure 3E). As soon as the NCPC (Figure 3F) reached 70% confluence, expansion and storage of these cells were performed. Cellular characterization was performed after the fourth passage. The NCPC showed expression for nestin and peripherin, an intermediate filament protein from proliferation cells and peripheral nervous system neurons, respectively (Figure 3G).

**FIGURE 4:**
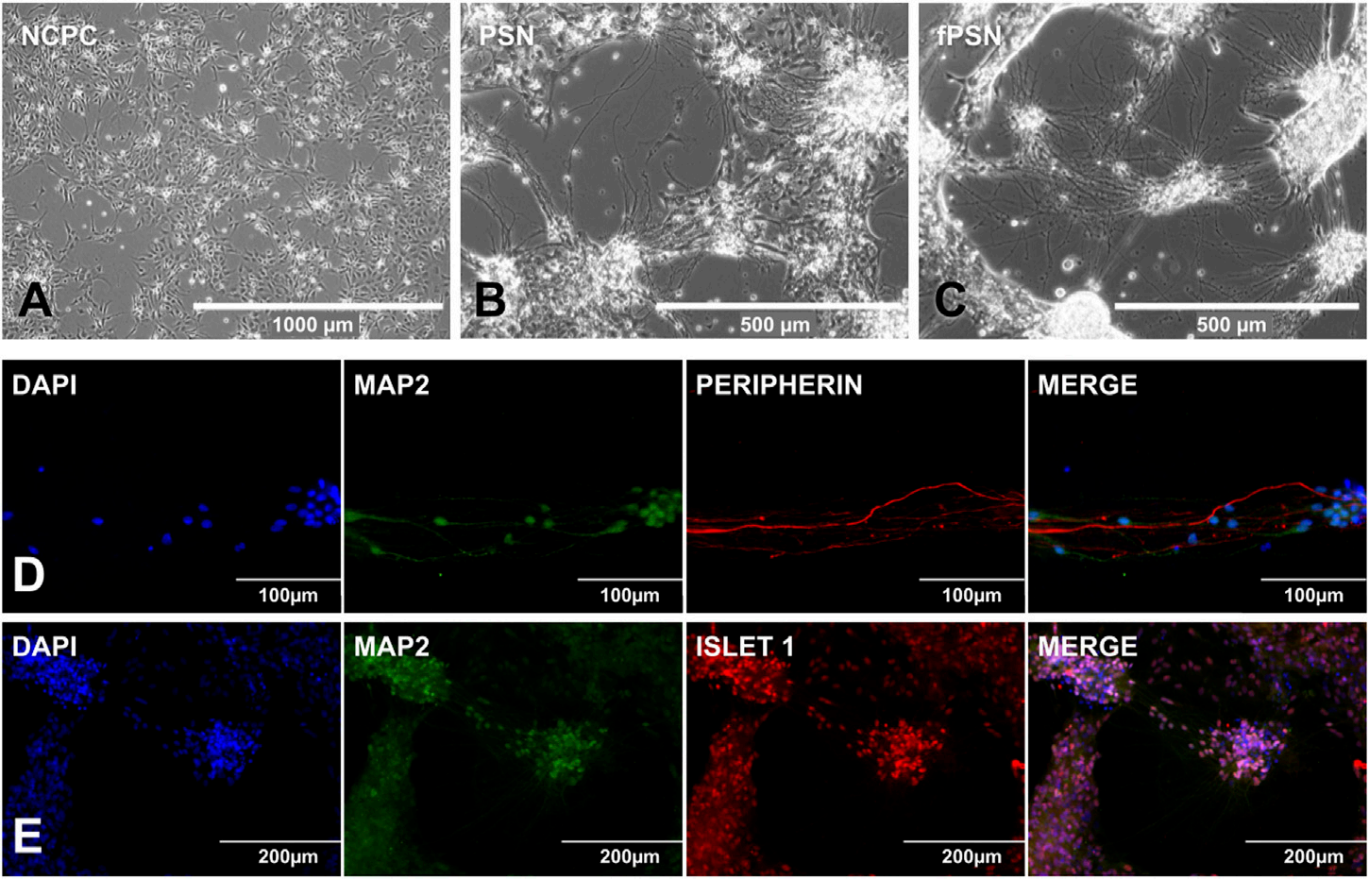
PSN differentiation and characterization. **(A)** NCPCs with ideal confluence to start PSN differentiation. **(B)** PSN after 20 days of differentiation. Cells showed a neuronal morphology with cells connected by cytoplasm extensions called neurites. **(C)** Functional PSNs (fPSNs) after 10 days of culture with the HEK-conditioned medium. fPSNs showed expression for MAP2, peripherin **(D)**, and ISLET1 **(E)**, confirming the protocol’s success. Scale bars: 1000 µm **(A)**, 500 µm **(B, C)**, 200 µm **(E)**, and 100 µm **(D)**.

From the NCPCs (Figure 4A), the PSN already assumed a neuronal morphology after 20 days of differentiation (Figure 4B), and, as expected, the neurite branches increased after the addition of HEK-conditioned medium during the last 10 days of the protocol (Figure 4C).

The protocol allowed the differentiation of fPSNs with long neurites of more than 300 μm. fPSN characterization was performed with MAP2, a neuron marker; peripherin (Figure 4D), a marker for peripheral nervous system neurons; and Islet 1 (Figure 4E), highly expressed in fPSNs but not in NCPCs (Guimarães et al., 2018), concluding that the cells obtained using this protocol are specific neurons from the peripheral system. In order to confirm the need to use the conditioned medium to allow the neurons to mature, three culture conditions were tested as follows: 1) using the HEK-conditioned medium during the last 5 days of the protocol; 2) using the HEK-conditioned medium during the last 10 days of the protocol, and 3) without the HEK-conditioned medium during the last 10 days of the protocol.

**FIGURE 5:**
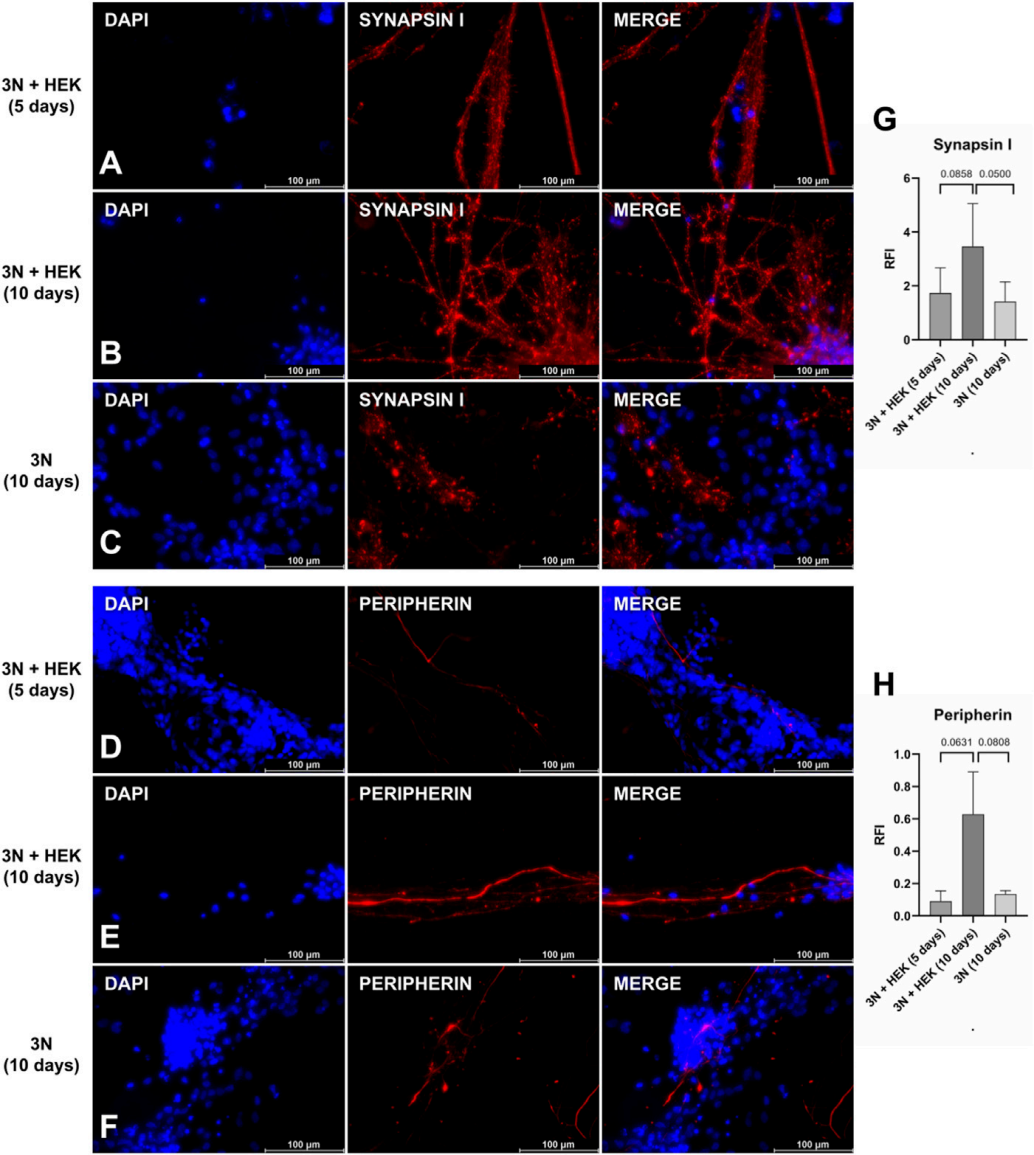
Peripheral sensory neurons under protocol modifications. Synapsin I **(A**–**C)** and peripherin **(D**–**F)** staining: after the application of the original protocol **(B** and **E)**, in which the last 10 days of culture contain 75% of the HEK-conditioned medium; with reduction of the final phase of the protocol to 5 days, maintaining the conditioned medium **(A** and **D)**, and keeping the 10-days protocol, but without the conditioned medium, only with 3 N medium **(C** and **F)**. Quantification of relative fluorescence intensity (RFI) of images stained with synapsin I **(G)** and peripherin **(H)** under protocol modification conditions (*p*-value represented at the top of the graph). A *p*-value above 0.05 is considered non-significant. Scale bar: 100 µm.

There was an increase in the number of pre-synaptic markers when 75% of the HEK-conditioned medium was used, in contrast to the protocol using only the 3 N medium (Figures 5B,C,G). Although a variation in the expression of peripherin appears, it is not significant when compared to the use of the HEK-conditioned medium for 5 or 10 days (Figures 5A,B,G). The expression of pre-synaptic markers does not vary significantly between the three culture conditions, although image analysis seems to show a subtle increase in the protocol’s original condition (using a conditioned medium) (Figures 5D–F,H). It lacks other evaluation methods to verify how sensitive this protocol is to changes.

## 5 Discussion

In this study, we established a protocol of differentiation to obtain NCPCs and fPSNs from iPSCs derived from SHED, aiming at their use in studies related to sensory disorders.

SHED were isolated from the dental pulp after exfoliation. Therefore, it is non-invasive, easy to obtain and isolate, and has an optimal application for studies, in which a patient’s skin biopsy would be impracticable and potentially traumatic, for example, in cases of pediatric diseases or syndromes.

This protocol is similar to the dual-SMAD inhibition method described by Chambers et al. (2012), in which what triggers neuronal differentiation are small inhibitory molecules such as LDN-193189, SB431542, and CHIR-99021, but this method and those with little modifications (Eberhardt et al., 2015; Schwartzentruber et al., 2018; Mis et al., 2019; Umehara et al., 2020) use fibroblast- or blood-derived iPSC lines. The other differentiation protocols for human sensory neurons, those that do not use this dual-SMAD method, start from hiPSCs derived from fibroblasts (Nickolls et al., 2020), from human embryonic stem cells (hESCs) (Bajpai et al., 2010; Schrenk-Siemens et al., 2014; Alshawaf et al., 2018; Hulme et al., 2020) or from murine fibroblasts (Wainger et al., 2014; Blanchard et al., 2015). We were the first group to establish this protocol using human SHED-iPSCs, whose cell source comes from accessible tissues.

This whole protocol takes around 2 months without going through a suspension culture stage, which includes three principal phases: NCPC induction, NCPC maintenance and expansion, and PSN induction and maturation; the latter is divided into two stages, one with only 3 N medium and supplements for 20 days and the other with the addition of the HEK-conditioned medium for 10 days, which seems to be essential for neuronal maturation (Guimarães et al., 2018). To start the protocol, iPSCs should have approximately 60% confluence composed of small colonies as failure to comply with this determination increases the risk of plate detachment, inadequate NCPC differentiation, and low densities in the initial plating in the dual-SMAD inhibition method, and promotes differentiation to neural crest cells (Chambers et al., 2009). In the first passages, NCPC differentiation is not fully established and may show slight changes in morphology and characterization, probably depending on the genetic background of the cell lineage.

Keratinocytes stimulate axonal growth in sensory neurons via the release of NGF and BDNF (Ulmann et al., 2007). In our protocol, these two factors were added exogenously, independent of the presence of the conditioned medium produced from the keratinocytes, and probably were sufficient to promote axonal growth despite the time and the presence of the conditioned medium. It would be interesting to test a co-culture model with sensory neurons and keratinocytes to improve PSN differentiation because recent studies have shown synaptic communication between them (Talagas et al., 2020).

Sensory neurons capture and transmit environmental or visceral stimuli to the CNS (Bear et al., 2016). In addition to the entire PNS, sensory neurons differentiate from neural crest cells during embryogenesis (Hall, 2009; Szobó& Mayor, 2018) from a portion of cells at the edge of the neural plate that starts to separate and migrate during the formation of the neural tube, leading to the origin of CNS cells (de Lahunta et al., 2016). Our protocol differentiates sensory neurons from somatic cells that also originated from the neural crest (Labau et al., 2022), and, as cells after reprogramming still carry their transcriptional signature, it is important that when working with iPSC technology, whenever possible, using the same embryonic origin of the cell source and the cell target of the study after the final differentiation is considered. An advantage presented is that the PSNs generated by this method do not present significant changes in their functionality according to variations in the final phase of the protocol, indicating that they are not as sensitive as those originating from fibroblasts (Schwartzentruber et al., 2018; Umehara et al., 2020), although additional analyses are necessary to confirm this result. Moreover, another benefit of using cells from the dental pulp of the exfoliated deciduous tooth is the low age of the donor, probably with fewer mitochondrial DNA mutations, which can alter the metabolism of the iPSCs (Kang et al., 2016).

This work showed the production of NCPCs and PSNs from SHED, cells with the same ontogenetic origin. Obtaining sensory neurons *in vitro* can open a wide variety of applications consistently, such as studies of pain or pruritus, the effects of peripheral inflammation and infections, drug testing, and altered sensitivity alterations in diseases or syndromes. Last, starting from the NCPCs established here, it is possible to develop new differentiation protocols to achieve other of its various cellular destinations.

## Data Availability

The original contributions presented in the study are included in the article, further inquiries can be directed to the corresponding author.
